# Wastewater surveillance and an automated robot: effectively tracking SARS-CoV-2 transmission in the post-epidemic era

**DOI:** 10.1093/nsr/nwad089

**Published:** 2023-03-31

**Authors:** Guanyong Ou, Yuxuan Tang, Shiyu Niu, Liwen Wu, Shaxi Li, Yang Yang, Jun Wang, Yun Peng, Chuanfu Huang, Wei Hu, Qinghua Hu, Yinghui Li, Yang Ping, Chao Lin, Boping Yu, Qi Han, Yabin Hao, Zhiguang Luo, Wende Tian, Han Zhang, Yingxia Liu

**Affiliations:** National Clinical Research Center for Infectious Disease, State Key Discipline of Infectious Disease, The Third People's Hospital of Shenzhen, Second Hospital Affiliated to Southern University of Science and Technology, Shenzhen 518112, China; School of Medicine, Southern University of Science and Technology, Shenzhen 518055, China; International Collaborative Laboratory of 2D Materials for Optoelectronics Science and Technology of Ministry of Education, Institute of Microscale Optoelectronics, College of Physics and Optoelectronic Engineering, Shenzhen University, Shenzhen 518060, China; National Clinical Research Center for Infectious Disease, State Key Discipline of Infectious Disease, The Third People's Hospital of Shenzhen, Second Hospital Affiliated to Southern University of Science and Technology, Shenzhen 518112, China; School of Medicine, Southern University of Science and Technology, Shenzhen 518055, China; National Clinical Research Center for Infectious Disease, State Key Discipline of Infectious Disease, The Third People's Hospital of Shenzhen, Second Hospital Affiliated to Southern University of Science and Technology, Shenzhen 518112, China; School of Medicine, Southern University of Science and Technology, Shenzhen 518055, China; National Clinical Research Center for Infectious Disease, State Key Discipline of Infectious Disease, The Third People's Hospital of Shenzhen, Second Hospital Affiliated to Southern University of Science and Technology, Shenzhen 518112, China; National Clinical Research Center for Infectious Disease, State Key Discipline of Infectious Disease, The Third People's Hospital of Shenzhen, Second Hospital Affiliated to Southern University of Science and Technology, Shenzhen 518112, China; National Clinical Research Center for Infectious Disease, State Key Discipline of Infectious Disease, The Third People's Hospital of Shenzhen, Second Hospital Affiliated to Southern University of Science and Technology, Shenzhen 518112, China; National Clinical Research Center for Infectious Disease, State Key Discipline of Infectious Disease, The Third People's Hospital of Shenzhen, Second Hospital Affiliated to Southern University of Science and Technology, Shenzhen 518112, China; Shenzhen Longhua Drainage Co., Ltd., Shenzhen 518060, China; Shenzhen Longhua Drainage Co., Ltd., Shenzhen 518060, China; Shenzhen Center for Disease Control and Prevention, Shenzhen 518055, China; Microbiology Laboratory, Shenzhen Center for Disease Control and Prevention, Shenzhen 518055, China; Power China Eco-Environmental Group Co., Ltd., Shenzhen 518102, China; Shenzhen Water Planning & Design Institute Co., Ltd., Shenzhen 518022, China; Shenzhen Academy of Environmental Sciences, Shenzhen 518001, China; School of Civil and Environmental Engineering, Harbin Institute of Technology (Shenzhen), Shenzhen 518055, China; Institute of Solid Wastes and Physical Environment Research, Shenzhen Academy of Environmental Sciences, Shenzhen 518001, China; Shenzhen Metasensing Technology Co., Ltd., Shenzhen 518000, China; Zhongmin (Shenzhen) intelligent ecology Co., Ltd., Shenzhen 518055, China; Research Centre for Eco-Environmental Sciences, Chinese Academy of Sciences, Beijing 100085, China; International Collaborative Laboratory of 2D Materials for Optoelectronics Science and Technology of Ministry of Education, Institute of Microscale Optoelectronics, College of Physics and Optoelectronic Engineering, Shenzhen University, Shenzhen 518060, China; National Clinical Research Center for Infectious Disease, State Key Discipline of Infectious Disease, The Third People's Hospital of Shenzhen, Second Hospital Affiliated to Southern University of Science and Technology, Shenzhen 518112, China

**Keywords:** SARS-CoV-2, wastewater surveillance, wastewater-based epidemiology, WWTP, automated robot

## Abstract

Wastewater-based epidemiology (WBE) has exhibited great utility in the early and rapid identification of SARS-CoV-2. However, the efficacy of wastewater surveillance under China's previous strict epidemic prevention policy remains to be described. We collected the WBE data of wastewater treatment plants (WWTPs) in the Third People's Hospital of Shenzhen and several communities to determine the significant effectiveness of routine wastewater surveillance in monitoring the local spread of SARS-CoV-2 under tight containment of the epidemic. The results of 1 month of continuous wastewater surveillance showed that positive signals for SARS-CoV-2 RNA were detected in the wastewater samples, and a significant positive correlation was observed between the virus concentration and the number of daily cases. In addition, the community's domestic wastewater surveillance results were confirmed even 3 days before, or simultaneously with, the infected patient being confirmed as having the virus. Meanwhile, an automated sewage virus detection robot, ShenNong No.1 robot, was developed, showing a high degree of agreement with experimental data, offering the possibility of large-scale multi-point surveillance. Overall, our results illustrated the clear indicative role of wastewater surveillance in combating COVID-19 and provided a practical basis for rapidly expanding the feasibility and value of routine wastewater surveillance for future emerging infectious diseases.

## Teaser

In the post-epidemic era, an automated wastewater surveillance robot exhibits clear effectiveness and value for pathogenic biomonitoring, paving the way for routine wastewater monitoring to prevent future emerging infectious diseases.

## INTRODUCTION

The coronavirus disease 2019 (COVID-19) pandemic, caused by a severe acute respiratory syndrome coronavirus 2 (SARS-CoV-2), is still spreading globally and causing high levels of patient mortality, especially with the emergence of multiple SARS-CoV-2 variants [1,2]. COVID-19 has been declared as a public health emergency of international concern (PHEIC) by the World Health Organization (WHO), which has affected more than 600 million people and caused more than 6 million deaths worldwide to date (World Health Organization, 2022).

Accumulating evidence suggests that SARS-CoV-2 is mainly transmitted through respiratory droplets and contacted fomites, and it can enter sewage through excrement, leading to the potential risk of fecal-oral transmission [3–5]. It has previously been observed that SARS-CoV-2 can be detected in feces after infection, which means that people with COVID‐19 pneumonia can be identified in time through excrement virus detection in the first 7–10 days of symptoms [6]. Yang *et al.* performed viral tests on environmental fomites from patients with COVID-19, and they found both symptomatic and asymptomatic patients with COVID-19 can potentially spread SARS-CoV-2 environmentally [7]. Furthermore, Wang *et al.* found that SARS-CoV-2 can still be shed in excrement for a period of time, even after the COVID-19 patients have recovered from the respiratory symptoms [8]. Meanwhile, the virus can survive for days in untreated wastewater [9,10]. The survival status of SARS-CoV-2 in the sewage, and the early warning and prediction of the epidemic based on the detection of nucleic acid fragments of the sewage virus WBE, have always been hot topics of concern [11–13]. A recent study showed that wastewater genomic surveillance can identify emerging virus variants up to 2 weeks earlier than detection through clinical genomic surveillance, and an omicron variant was detected in wastewater 11 days before the first clinical report in San Diego [12]. In addition, a case report in Hong Kong, China, also revealed that WBE for COVID-19 was highly effective in identifying buildings affected by SARS-CoV-2 [14]. Actually, wastewater surveillance based on WBE has been successfully and extensively used abroad in the preclinical identification of various viruses such as poliovirus [15] and norovirus [16,17], so as to establish WBE surveillance networks. Thus, it is essential to promote wastewater surveillance of various virus pathogenies and establish WBE surveillance systems, which can greatly save time and also medical care costs, contributing to the preclinically rapid identification of SARS-CoV-2 and early warning for nearby communities. However, it was poorly characterized and understood whether the WBE surveillance system will still be of significant benefit under China's previous tight epidemic prevention policies, especially in a situation like Shenzhen where almost all citizens are routinely tested for COVID-19 on a daily basis.

**Figure 1.**
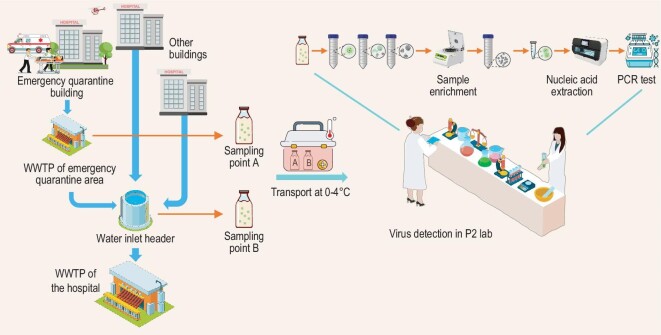
Information of sampling locations in the two WWTPs.

In this contribution, we investigated the WBE data collected from the wastewater treatment plant (WWTP) in The Third People's Hospital of Shenzhen and the corresponding emergency hospital area (where COVID-19 patients were quarantined) (Fig. 1), to assess the feasibility and value of rapid scale-up of routine wastewater surveillance sampling.

## RESULTS

### Recovery rate and limit of detection using the aluminum hydroxide adsorption–precipitation method

**Figure 2.**
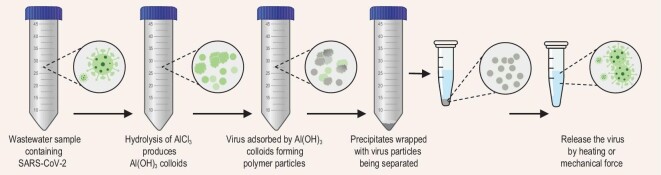
The principle of aluminum hydroxide adsorption–precipitation method.

In this work, virus enrichment was carried out using the aluminum hydroxide adsorption–precipitation method and the principle of aluminum hydroxide adsorption–precipitation is presented in Fig. 2. The recovery rate is shown in Table S1. A standard curve was obtained by PCR test using quantitative pseudovirus. To test the recovery rate of this method and ensure the universality of the experimental results for various types of sewage, wastewater collected from 3 different regions was selected for parallel experiments. Below 200 copies/mL spiking concentration, the average recovery rate was between 10% to ∼60% for N target while only 1.6% to ∼28% for the Orf1ab target. In order to explore the performance of this method at extremely low concentrations, wastewater samples with much lower concentration of pseudovirus loads were enriched and PCR-tested at very low concentrations of 1.25, 3.13, 6.25, and 12.5 copies/mL. To verify the repeatability of the method, the occurrence of a positive signal out of 100 experiments at 4 low concentrations was recorded, and for each experiment 3 parallel tests were carried out to guarantee the reliability of the results according to the national standard WS/T 799–2022. As shown in Table S2, the method used in this work is very feasible even at a concentration as low as 6.25 copies/ml (74%) or ∼1.56 copies/ml (21%), providing a possibility to detect positive sewage samples covering a large upstream population. It is noticed that the N target presents a more robust and valid signal than the Orf1ab target, which is consistent with some previous reports [18,19]. Therefore, the N target is selected as the representative target in this work.

### SARS-CoV-2 RNA in hospital wastewater samples

From August 10, 2022 to September 9, 2022, wastewater samples were collected in the WWTP of area A (the emergency quarantine area) and B (the whole hospital area), 3 times a day (8 am, 1 pm, 6 pm, which was defined as 1, 2, 3, respectively). It is worth noting that the wastewater samples were absent from September 3 to September 5 due to the closure and administration stages of the COVID-19 epidemic at that time. The numbers of daily clinical confirmed cases and new cases in the hospital were 117 and 8, respectively, at the beginning of the present study (August 10, 2022), and all confirmed and new cases were quarantined at the emergency quarantine area.

**Figure 3.**
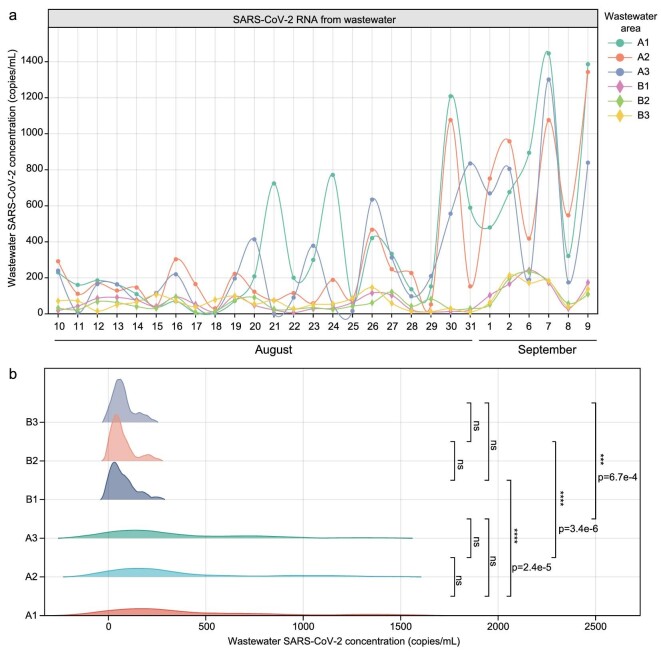
Detection of SARS-CoV-2 RNA from wastewater in hospital. Daily SARS-CoV-2 RNA (‘N’ target) concentration from August 10, 2022 to September 9, 2022 in wastewater collection areas A and B (a). The differences in SARS-CoV-2 RNA concentration between area A and B (b). Area A: the converging area of the wastewater from emergency hospital; area B: the converging area of all the wastewater from whole hospital. Numbers indicate when samples were collected, with 1 for 8 am, 2 for 1 pm, and 3 for 6 pm.

For 1-month detection, the higher concentrations of SARS-CoV-2 RNA were obviously more observable in wastewater samples in area A than in area B (Fig. 3a and b), though both areas had positive signals for SARS-CoV-2 RNA in all tested wastewater samples. Further, through comparing the detection differences in SARS-CoV-2 RNA at different collection times of wastewater samples in a single day, we found that there was no significant difference on sample collection time (Fig. 3b), indicating that wastewater samples can be collected at any time of day.

### The association between wastewater SARS-CoV-2 RNA concentration and clinically reported cases

**Figure 4.**
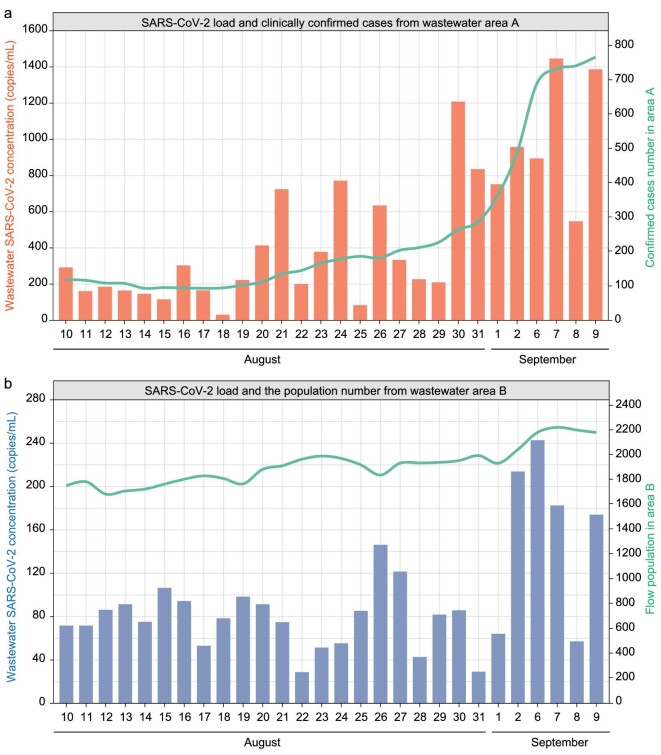
The distribution of daily SARS-CoV-2 RNA concentration with clinical confirmed cases (a) and daily flow number (b) from August 10, 2022 to September 9, 2022 in wastewater collection areas A and B.

The hospital accommodates approximately 5000 flow population (including hospital staff) and 1000 inpatients per day. We assumed that one-tenth of the flow population defecated in the hospital toilet, and then assessed the daily flow number of defecations in the whole hospital. The distribution changes of SARS-CoV-2 RNA concentration in wastewater with daily confirmed cases and flow numbers in areas A and B from August 10, 2022 to September 9, 2022 are shown in Fig. 4.

**Figure 5.**
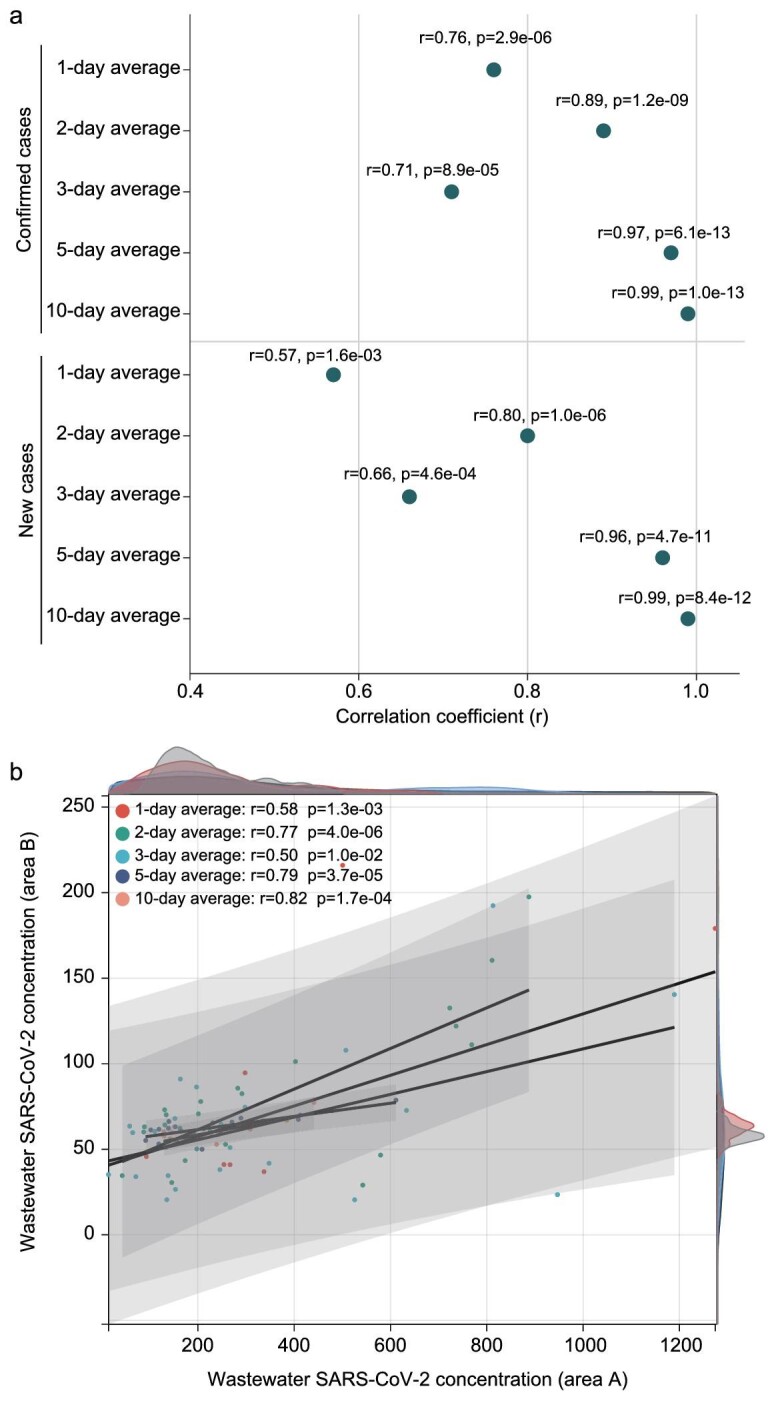
Correlation of SARS-CoV-2 RNA concentration in wastewater with clinical reported cases. Correlation of 1-, 2-, 3-, 5-, and 10-day average between the SARS-CoV-2 RNA concentration and daily clinical reported cases (including confirmed cases and new cases) in area A (a). Correlation of 1-, 2-, 3-, 5-, and 10-day average SARS-CoV-2 RNA concentration in area A and area B (b).

To further investigate the association between wastewater SARS-CoV-2 RNA concentration and clinically reported cases, in addition to considering the normal distribution of the data, Pearson's correlation analysis was employed to assess the correlation. The observed concentrations of SARS-CoV-2 RNA in wastewater samples from area A were positively correlated with the presence of clinically reported cases (Fig. 5a). And the strongest correlation between the SARS-CoV-2 RNA concentrations in wastewater and the numbers of clinically reported cases, including daily confirmed cases (r = 0.99, p = 1.0e-13) and new cases (r = 0.99, p = 8.4e-12), was observed at 10-day average wastewater concentrations (Fig. 5a). Meanwhile, it is worth noting that, in a 1-day average, SARS-CoV-2 RNA load in wastewater exhibited a strong positive correlation with the presence of daily confirmed cases (r = 0.76, p = 2.9e-06) and daily new cases (r = 0.57, p = 1.6e-03) (Fig. 5a). In addition, SARS-CoV-2 RNA concentrations in wastewater from area A were positively correlated with that of area B (Fig. 5b), and the strongest positive correlation was also observed at 10-day average wastewater concentrations (r = 0.82, p = 1.7e-04) (Fig. 5b).

Through investigating the association between sewage virus concentrations and clinically confirmed cases in area B, we found that the wastewater SARS-CoV-2 RNA concentrations were also positively correlated with the number of clinically confirmed cases (r = 0.64, p = 2.7e-04) (Fig. S1a). Taking into account the slight change in daily flow population, which can be thought of as a small community, we further explored the potential association of wastewater virus concentration with the ratio of clinically confirmed cases to the daily flow population. The results showed that there was a strong positive correlation between the wastewater SARS-CoV-2 concentrations and the ratio of confirmed cases in the population (r = 0.64, p = 2.5e-04) (Fig. S1b), suggesting a positive correlation between the infected population in the community and the virus concentrations in the sewage.

### The detection and inspiration of positive sewage samples in Longhua District

**Figure 6.**
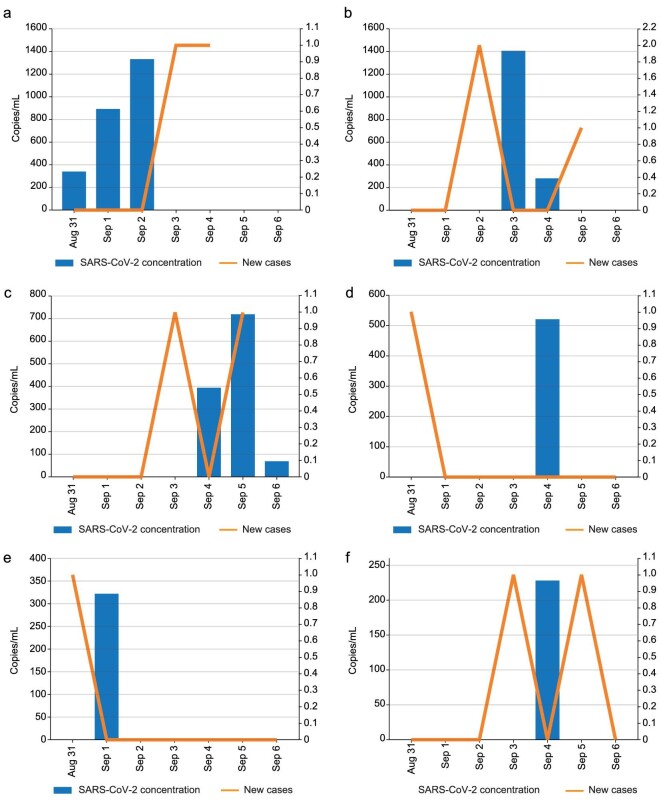
Sewage detection results in a week of DQXC-01 (a), FQXCOO1-4 (b), WYNJD001 (c), ZK-01 (d), ZK-03 (e), MLXC-01 (f) (blue column) and confirmed cases in upstream communities (orange line).

The actual SARS-Cov-2 loaded in domestic sewage is much lower than that in the Third People's Hospital of Shenzhen, especially under the previous strict epidemic prevention control in the city. Residents in Shenzhen are required to conduct throat swab testing at least every 2 days, and transfer immediately once being confirmed infected, while residents who are in close contact with them but do not test positive remain in the residence for isolation and observation. In this work, continuous sewage virus monitoring was carried out in the medium- and high-risk areas where an infection has occurred, and it was found that the positive status of sewage virus was almost synchronous with or up to 3 days delay to that of throat swabs (Fig. 6b-f). For the result at Dongquan New Village (sampling spot DQXC-01), it was even found that the sewage was positive 3 days in advance than confirmed cases published by the health department (Fig. 6a), which may be due to the fact that throat swab detection largely depends on the subjective will of residents, or if there was an error in the sampling operation such as the swab not accurately scratching the oral mucosa, or other experimental errors. It is noted that even if the patient is transferred immediately after the diagnosis, the excreta of the positive patient will usually remain in the sewage pipe for about 3 days, making it difficult to judge whether the positive sewage is caused by the confirmed cases that have been transferred or by new cases.

### The detection robot for SARS-CoV-2 in wastewater

An automated solution for wastewater SARS-CoV-2 detection is highly demanded due to the complexity and long time consumption of manual laboratory work. A detection robot named ShenNong No.1 (Shenzhen Metasensing Co., Ltd) was then used for fast detection (Fig. S2) and, due to its simplicity and adjustability, the aluminum hydroxide adsorption–precipitation method was applied to the robot. To test the performance of ShenNong No.1, six wastewater samples collected from the Third People's Hospital of Shenzhen were detected by this robot with a comparison of manual tests. Each sample (total volume 300 mL) is tested three times (40 mL per test) for the whole process, and the coefficient of variation (CV) of Ct value was measured. With almost consistent Ct results, the robot showed higher consistency of multiple tests on the same water sample and also behaved more agilely (Table S3).

## DISCUSSION

The ongoing COVID-19 pandemic caused by SARS-CoV-2 was considered to be transmitted from person to person via droplet infections and fecal-oral transmission [5,20,21]. Despite international efforts to contain the spread of SARS-CoV-2, it continues to be a serious global health challenge. In particular, virus shedding and transmission in patients with long incubation periods and asymptomatic infections, as well as in pre-symptomatic infections [6,7,22] and virus mutations add to the burden of outbreak prevention and management [23,24]. However, clinical monitoring of each individual in a community is impracticable and quite expensive on an uninterrupted basis. Also, the presence of asymptomatic patients makes it impossible to fully cover the community with regular clinical testing in a cheap way. Hence, it is undeniable that additional testing of close contacts is essential to identify and isolate potentially infected individuals and to prevent further transmission. Environmental wastewater surveillance, on the contrary, can provide an ongoing, economical way to keep track of local communities for outbreaks and subsequent propagation [25,26]. Actually, WBE has proven to be a promising tool for rapid identification of the regions with SARS-CoV-2 carriers early in transmission and monitoring virus distribution while protecting anonymity [27]. However, little is known about whether WBE data still accurately reflect early transmission under the government's tight COVID-2019 epidemic management.

Herein, 1-month wastewater surveillance for SARS-CoV-2 demonstrated a strong correlation between virus concentration in wastewater and the presence of confirmed cases. Furthermore, the strong correlation between virus concentrations upstream and downstream of the wastewater station also indicated that the virus spread over time. An additional valuable point is that since there is no significant regularity in the patient's detoxification time, community sewage monitoring should, as much as possible, have a certain sampling frequency to cover the whole day, otherwise the feces of some patients may be missed.

The pilot project in Longhua District, Shenzhen, shows that the sensitivity of sewage monitoring is similar to that of throat swab detection. This provides a new perspective that through reasonable complementation and adjustment, such as the way adopted by the Hong Kong government, to conduct mandatory throat swab detection in the upstream area after sewage detection is positive, can minimize government costs while ensuring that the sources of infection can be found. Other virtues such as reduction of medical waste disposal caused by numerous throat swab tests, alleviation of residents queuing for the nucleic acid test, or even early warning by its nature of passive detection in some cases, makes it much more advanced, reliable, and recommendable.

The automatic solution opens a new avenue for fast and online detection of SARS-COV-2 in wastewater, along with its high consistency and convenience. Virus signals can be easily missed due to inadvertent error in manual experimental operation, because the virus content in domestic sewage is extremely low. Fine and sophisticated automation operations can help to avoid deviation and improve the accuracy of the overall system.

In summary, our 1-month wastewater surveillance study showed a significant linear correlation between wastewater virus concentrations and the presence of clinically reported cases, suggesting that even under strict government epidemic prevention and management, wastewater data continue to exert significant preventive effectiveness and affordability in combating COVID-19 or future infectious disease outbreaks.

## MATERIALS AND METHODS

The whole procedure follows the national health industry standard WS/T 799–2022, method for the enrichment and nucleic acid detection of SARS-CoV-2 in sewage, including procedures of sample collection, sample enrichment, nucleic acid extraction, and viral detection. All the experiments were carried out in the P2 biosafety laboratory. Detailed methodological data can be found in the online supplementary files.

### Sample collection

The instantaneous sampling method was adopted in this work. In the Third People's Hospital of Shenzhen, wastewater from two spots, one is the influent of  WWTP in the emergency quarantine area and the other is the main WWTP of the whole hospital (Fig. 1), were collected in a time scale of 1 month.

### Sample enrichment

Three methods for wastewater enrichment are recommended by the standard WS/T 799–2022 and the aluminum hydroxide adsorption–precipitation method was used in this work due to its simplicity and lower cost. Briefly, 30 min centrifugation of 50 mL water sample was first applied to remove suspended solids (4°C, 2500 g) and then 0.5 mL of AlCl3 solution (0.3 mol/L) was added to the supernatant fluid with pH adjusted to 6.0 to allow aluminum hydroxide colloid emerging and adsorbing the virus. After that the pellet containing virus particles was separated by 5 min centrifugation (4°C, 1900 g) and 0.2 g EDTA-2Na was then mixed with the pellet to get higher releasing efficiency and to ensure the stability of the RNA [28].

### Nucleic acid extraction

The Nucleic Acid Extraction Kit (Magnetic Beads Method, Zybio) was used for viral extraction following the manufacturer's protocol.

### Viral detection and quantification

The 2019-nCoV Nucleic Acid Detection Kit (Wuhan EasyDiagnosis Biomedicine Co., Ltd) was used for viral detection and quantification following the manufacturers instructions, with N and Orf1ab target primers. Reverse transcriptase quantitative polymerase chain reaction (RT-qPCR) was used for the detection of viruses. The quantitative pseudovirus used for standard curve plotting and the positive control sample used for reference of enrichment, extraction, and PCR detection were all purchased from BDS company in this work, and the negative control samples were nuclease-free water.

### The recovery rate

The recovery rate (R_r_) is defined as the ratio of the RNA concentration recovered in the concentrated solution after enrichment (C_enri_) to the RNA concentration in the initial sewage sample before enrichment (C_inti_), as shown in equation 1. The RNA concentration (C_RNA_) is determined by the Ct value of RT-PCR standard curve of quantitative pseudovirus (equation 2).


(1) \begin{eqnarray*}{{\rm{R}}}_{\rm{r}} = \frac{{{{\rm{C}}}_{{\rm{enri}}}}}{{{{\rm{C}}}_{{\rm{inti}}}}} \times 100\% \end{eqnarray*}

(2) \begin{eqnarray*} {{\rm{C}}}_{{\rm{RNA}}} = f\left( {{\rm{Ct}}} \right)\end{eqnarray*}

### Virus detection using ShenNong No.1 robot

The ShenNong No.1 robot contains several units, including automatic sewage sampling, automatic enrichment and concentration, automatic nucleic acid extraction, and automatic PCR detection. Samples were manually placed on the pallet then the whole process was accomplished automatically by the robot.

### Statistical analysis

Statistical analyses were performed in Excel, SPSS 25.0 and R v 4.0.2. A statistical significance level of *p* < 0.05 was considered for all tests.

## Supplementary Material

nwad089_Supplemental_Files
