# Supplementary material for: Wastewater surveillance and an automated robot: effectively tracking SARS-CoV-2 transmission in the post-epidemic era
Source: Natl Sci Rev. 2023 Mar 31;10(6):nwad089. doi: 10.1093/nsr/nwad089 (PMC10171627; doi:10.1093/nsr/nwad089)
Supplement: nwad089_Supplemental_Files [file nwad089_supplemental_files.zip › Supplementary data.pdf]

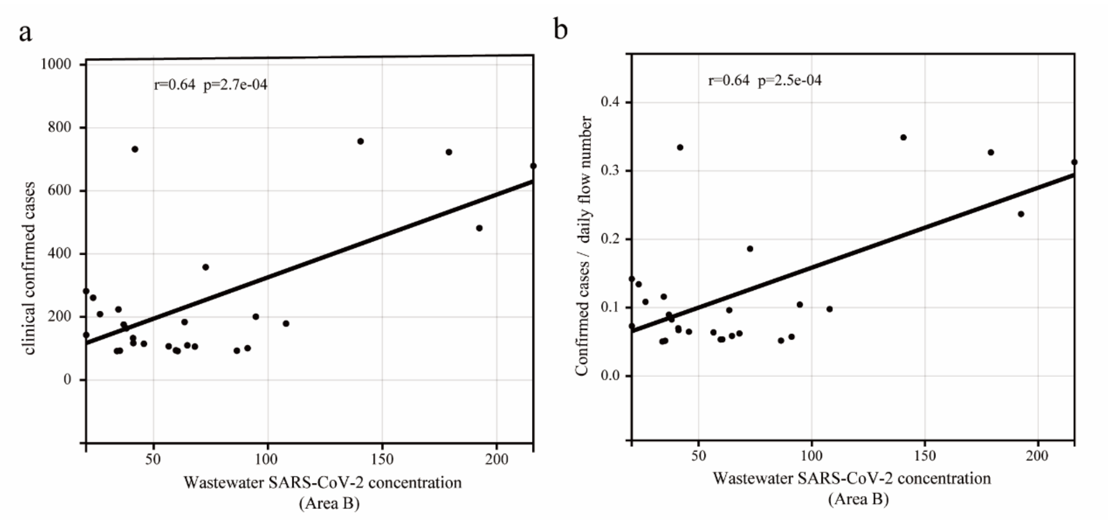

Figure S1. Correlation of wastewater SARS-CoV-2 concentration with confirmed clinical cases (**a**) and ratio of confirmed cases to daily flow number (**b**) in area B.

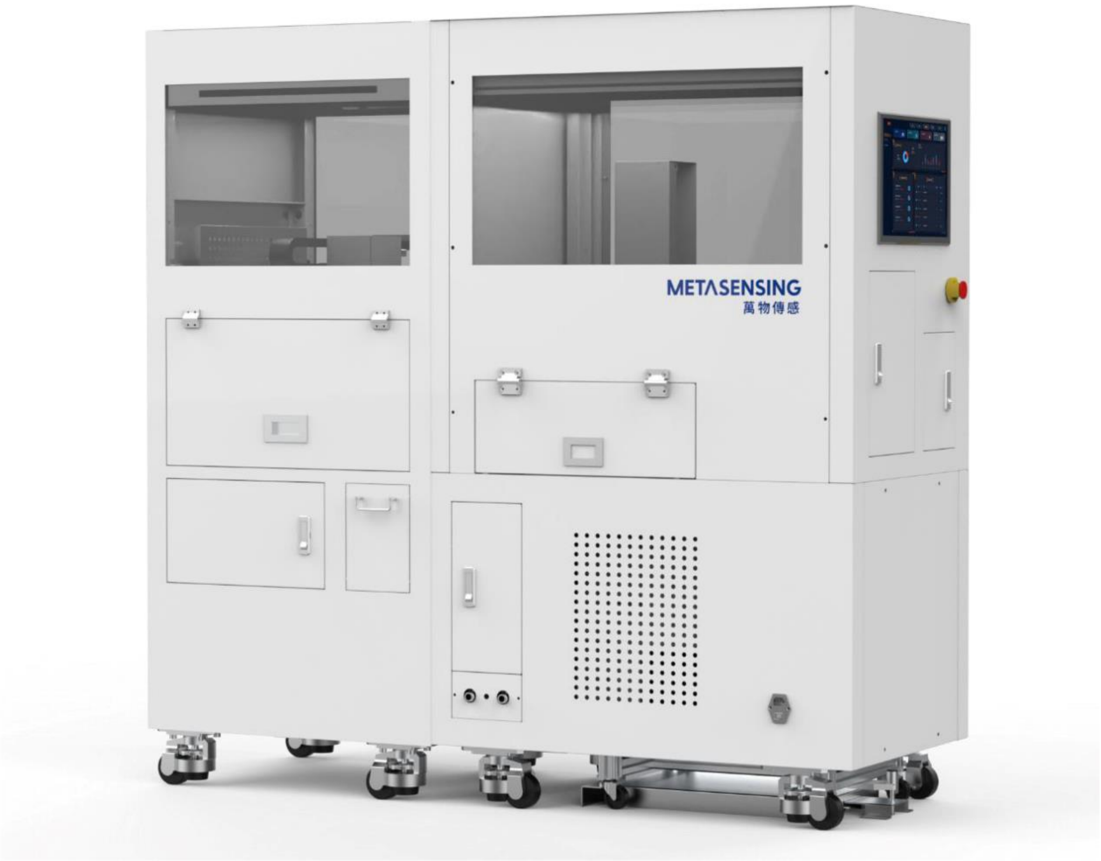

Figure S2. The wastewater SARS-COV-2 detection robot ShenNong No.1

Table S1. The recovery rate at various concentrations between 6.25-200 copies/ml

Concentration (copies/ml)	Recovery Rate for sewage 1		Recovery Rate for sewage 2		Recovery Rate for sewage 3	
(target)	N	Orf1ab	N	Orf1ab	N	Orf1ab
200	29.02%	7.08%	26.42%	7.14%	87.35%	9.71%
100	13.33%	2.71%	16.75%	2.78%	32.62%	3.12%
50	5.07%	1.63%	15.25%	1.69%	21.75%	4.45%
25	16.68%	2.31%	11.38%	2.40%	12.09%	2.94%
12.50	24.31%	1.64%	12.49%	1.71%	7.16%	4.27%
6.25	50.03%	15.94%	45.22%	16.47%	53.24%	28.27%

Table S2. The positive rate at concentration of 1.56 copies/ml, 3.13 copies/ml, 6.25 copies/ml, 12.50 copies/ml, 100 tests were carried at each concentration

Concentration (copies/ml)	positive rate (out of 100 times)	
(target)	N	Orf1ab
12.50	92	78
6.25	74	56
3.13	38	19
1.56	21	4

Table S3. Comparison of tests by human and ShenNong No.1 robot on 6 wastewater samples.

Sample No.	PCR results		C.V	
	Manul	Robotic	Manul	Robotic
Sewage 1	29.11	29.32	2.02%	0.20%
	29.46	29.43		
	28.32	29.41		
Sewage 2	29.48	28.14	2.79%	1.54%
	28.04	27.50		
	28.17	27.33		
Sewage 3	26.90	25.81	3.58%	1.20%
	28.51	26.34		
	26.74	25.79		
Sewage 4	30.07	30.10	2.43%	1.35%
	28.85	30.92		
	30.13	30.47		
Sewage 5	34.31	34.46	2.97%	0.40%
	35.33	34.44		
	36.41	34.69		
Sewage 6	30.92	31.26	2.22%	1.15%
	32.31	31.58		
	31.47	30.86		

Supplementary methods

Sample collection

The instantaneous sampling method was adopted in this work. In the Third People's Hospital of Shenzhen, wastewater from two spots, one is the influent of WWTP in the emergency quarantine area and the other is the main WWTP of the whole hospital (Fig. 1), were collected in a time scale of one month. For each spot, 300 mL of wastewater was collected 3 times a day (8 am, 1 pm, 6 pm, which was defined as 1, 2, 3, respectively). As for the medium and high-risk areas in Longhua district, the 300 mL sewage sample of each building was collected at 8 am per day lasting for one week. All the samples were inactivated on site and then delivered on ice to the lab within 3 hours.

Nucleic acid extraction

The Nucleic Acid Extraction Kit (Magnetic Beads Method, Zybio) was used for viral extraction following the manufacturer's protocol. First, 15 μL protease k is added into 200 μL solution of the concentrated wastewater sample then react at 65 ℃ for 4 minutes by adding 500 μL lysis solution and 4 μL magnetic beads solution. Second, nucleic acid fragments in the mixed solution are magnetically adsorbed for 1 minute and then washed by 600 μL detergent for 30s. Finally, after magnetic adsorption for 1 minute, the nucleic acid is eluted with 100 μL eluent at 80℃ for 2 minutes, and then the supernatant containing RNA is obtained after the magnetic beads being transferred.

Viral detection and quantification

Each PCR test takes 5 μL of RNA sample with 200 μL of PCR reaction reagent. First, the RNA undergoes reverse transcription at 50℃ for 15 minutes and pre-denaturation at 95℃ for 30 seconds. Then it goes through 40 cycles, each of which contains denaturation at 95℃ and annealing extension at 60℃ for 40 seconds. The fluorescence signals of N target, ORF1ab target and internal reference target RNaseP are detected after each cycle. The quantitative pseudovirus used for standard curve plotting and the positive control sample used for reference of enrichment, extraction and PCR detection were all purchased from BDS company in this work, and the negative control samples were nuclease-free water.
