# Supplementary material for: Wastewater surveillance and an automated robot: effectively tracking SARS-CoV-2 transmission in the post-epidemic era
Source: Natl Sci Rev. 2023 Mar 31;10(6):nwad089. doi: 10.1093/nsr/nwad089 (PMC10171627; doi:10.1093/nsr/nwad089)
Supplement: nwad089_Supplemental_Files [file nwad089_supplemental_files.zip › Teaser text.docx]

**Teaser text**

In the post-epidemic era, automated wastewater surveillance robot exhibits clear effectiveness and value for pathogenic biomonitoring, paving the way for routine wastewater monitoring to prevent future emerging infectious diseases.
